# Factors affecting ethnobotanical knowledge in a mestizo community of the Sierra de Huautla Biosphere Reserve, Mexico

**DOI:** 10.1186/1746-4269-10-14

**Published:** 2014-01-27

**Authors:** Leonardo Beltrán-Rodríguez, Amanda Ortiz-Sánchez, Nestor A Mariano, Belinda Maldonado-Almanza, Victoria Reyes-García

**Affiliations:** 1 Facultad de Ciencias Biológicas, Universidad Autónoma del Estado de Morelos, Av. Universidad Nº 1001, Cuernavaca CP: 62210, Mexico; 2 Present address: Postgrado en Botánica, Colegio de Postgraduados, Montecillo, Texcoco C.P. 56230, México; 3 Centro de Investigación en Biodiversidad y Conservación (CIByC), Av. Universidad Nº 1001, Cuernavaca CP: 62210, Mexico; 4 ICREA and Institut de Ciencia i Tecnologia Ambientals, Universitat Autónoma de Barcelona, 08193 Bellatera, Barcelona, Spain

**Keywords:** Ethnobotanical knowledge, Socio-economic variables, Mestizo community, Mexico, Useful plants

## Abstract

**Background:**

Worldwide, mestizo communities’s ethnobotanical knowledge has been poorly studied. Based on a mestizo group in Mexico, this study assesses a) the use value (UV) of the local flora, b) gendered differences in plant species, and c) the association between socio-economic variables and ethnobotanical knowledge.

**Methods:**

To assess the degree of knowledge of plant resources, we conducted 41 interviews collecting information on knowledge of local plant resources and the socio-economic situation of the informant. We also collected free listings of useful plants by category of use to identify the UV of each species. With the support of key informants, we photographed and collected the plant material recorded during the interviews and free listings on five different habitats. Paired t-tests and a Wilcoxon signed rank test were used to determine differences in the number of species known by men and women. Differences in distribution were analyzed by means of the Shapiro–Wilk’s W normality tests. To determine the association of socio-economic factors and ethnobotanical knowledge, we used a non-metric multidimensional scaling analysis (NMDS).

**Results:**

Informants listed 185 species. Medicinal plants constituted the most diverse group (90 species). Tropical deciduous forest is the habitat that concentrates the highest proportion of plant resources (80 species). The use-values were classified into three groups: A (4–6 UV; three species), B (0.35-1.37 UV; 39 species) and C (0–0.29 UV; 143 species). High-quality wood species and those associated to religious ceremonies had the highest UV. Women’s and men’s knowledge of plant species showed statistically significant differences at the interspecific and the intracategorical levels (Student’s test, T15 = 4.8, p < 0.001). Occupation, gender and age were statistically significant associated to ethnobotanical knowledge (p < 0.05), whereas income, education level, and place of origin were not.

**Conclusion:**

This research improves our understanding of the socio-economic activities associated with the intracultural distribution of ethnobotanical knowledge among mestizo Mexican communities. It also provides information on plant resources and habitats and how local peasants value them. This information could help in the development of proposals to improve biocultural conservation and strengthen traditional knowledge systems for effective forest management.

## Background

Traditional knowledge, understood as cumulative body of knowledge, practices and beliefs about the environment evolving by adaptive processes and handed down through generations by cultural transmission [1,2], has been widely documented in diverse Mesoamerican groups. Most of this research has focused on indigenous communities located in Mexico. In Mexico, studies have approached different fields of traditional knowledge including the domestication of plants [3,4], the folk classification of the natural world [5], the cultural meaning of wild species [6], the loss and changes of knowledge [7,8], the use and management of wild species, e.g., food, timber, textiles, fuels and others [9,10], particularly the knowledge of medicinal plants [11].

It has been argued that the use of plants in indigenous communities is associated to biological, ecological and socio-cultural factors, including production techniques and practices, religion, gender, and age [12-16]. Such aspects have been extensively studied in several indigenous societies in other parts of the world [12-14,17], highlighting the different patterns in knowledge distribution and loss, leading to different changes in the use and management of such resources. However, researchers have paid scant attention to the association between such socio-cultural and socio-economics factors and the acquisition of traditional knowledge in mestizo communities see [15,18,19], for some exceptions.

The study of the traditional knowledge of mestizo communities is important because such communities account for about 75% of Mexico forested surface [20], so understanding the process of knowledge acquisition in those communities, as well as what are the species with larger use value can have important implications for the conservation and management of forested resources [21]. An additional reason to focus on mestizo communities is that their knowledge seems to be different from that of indigenous societies. Although some studies in Mexico do not show significant differences between indigenous and mestizo communities [22], other studies suggest that indigenous communities use more frequently medicinal, edible, and firewood plant species, whereas mestizo communities use more frequently plant species for construction [23].

Previous ethnobotanical studies carried out with mestizo groups in Mexico have been mainly oriented to making inventories of useful plants at specific locations [24], although the focus has recently changed and nowadays researchers are more interested in examining how ecological and social aspects (i.e., occupation, education level, gender, degree of urbanization, or relation with other communities) shape traditional knowledge [22,25-27].

In this context, we conducted a research in the mestizo community of El Salto, Morelos, Mexico. The research aimed to answer the following questions: 1) Which plant species do mestizo people use to satisfy their needs? 2) Which is the use-value of the local flora? 3) Are socio-economic variables associated to the acquisition of ethnobotanical knowledge in a mestizo community?.

Researchers have previously studied the association between ethnobotanical knowledge and socio-economic factors. Among the factors previously studied researchers have focused on the age [12,27-30], sex [6,12] 115 [29-31], the educational level [15,25,30], origin [32,33], and the occupation and the wealth [34,35] of informants. Among those, researchers have found that those having a stronger influence on shaping ethnobotanical knowledge distribution are age, sex, education level and wealth.

For example, several studies have found a positive association between age and traditional ethnobotanical knowledge [12], although some other studies have not found such association [15]. In contrast, the differences in ethnobotanical knowledge between men and women seem to be more consistent, with studies finding that men have a larger knowledge than women [13,15,29,31], although the trend seems to be inverse in relation to medicinal plants [30]. Such differences are generally explained by sexual distribution of work [6,36]. Some research also suggest that ethnobotanical knowledge decreases with the increase of education [7,25,27,37] and wealth [34,35]. Several of those characteristics are also linked to the process of acculturation and the loss of indigenous languages (among indigenous communities) [7,27,37]. Some studies highlight the importance of occupation on traditional knowledge [25,38]. Martínez-Ballesté *et al.*[25] find that larger involvement in agricultural activities resulted in a loss of traditional ecological knowledge, as a consequence of the environmental transformation and loss of biodiversity. In contrast, those activities more dependent on the natural environment are associated to maintenance of traditional knowledge.

Given those previous findings, we hypothesize that the distribution of traditional knowledge will be patterned across socio-economic characteristics. Specifically, we expect to find that men, older people, people born in the area, and poorer people will have higher levels of traditional knowledge than people without those characteristics. We also hypothesize that people whose occupation depends on the environment, like people who practice extensive agriculture and stockbreeding, might also have larger levels of traditional knowledge.

Understanding the dynamics of ethnobotanical traditional knowledge among mestizo groups will provide information that is relevant to ethnobotany in two ways. First, it will help to understand the relations between social processes and the use and management of plant resources. And second, it will enable the development of approaches to conserve wild species taking into account the patterns of plant knowledge based on the use value of plant resources. At the applied level, the traditional knowledge of mestizo communities constitutes one of the multiple manifestations of Mexico’s cultural diversity, and it is considered to be of great importance in terms of biological conservation both nationally and internationally [39,40]. It is also relevant for the development of ecologically and economically feasible proposals of socially just rural development, aimed to the promotion of bio-cultural conservation [2,41].

## Methods

### Study area

**Figure 1:**
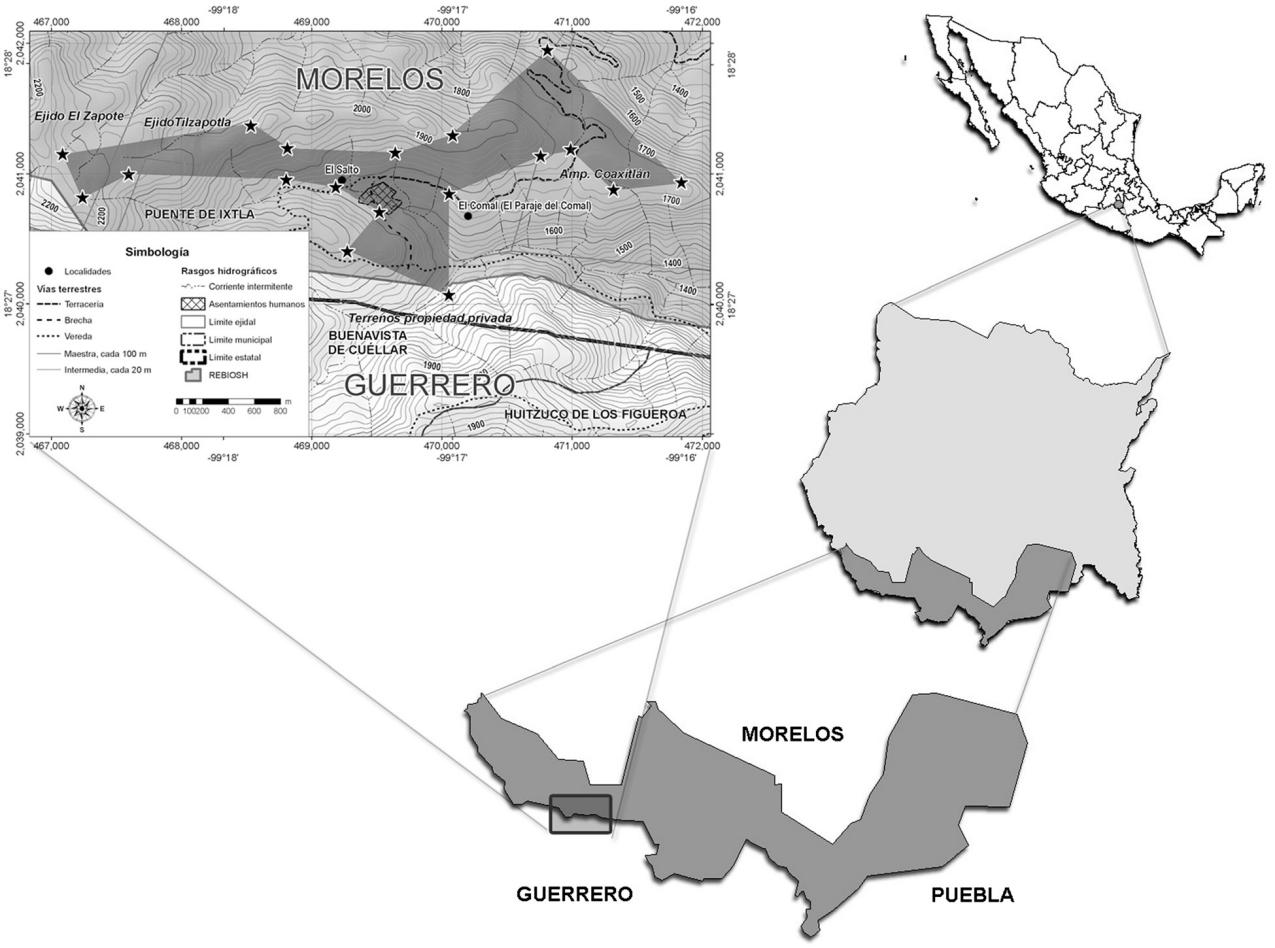
Location and boundaries recognized by the people of El Salto, Puente de Ixtla, Morelos, Mexico.

This study was carried out at El Salto, a rural community located in the southern area of the state of Morelos, Mexico, within the Biosphere Reserve Sierra de Huautla. Its territory belongs to the mountain range known as Sierra Madre del Sur and it is part of the northern extreme of the Sierra de Huitzuco in the state of Guerrero, known as Cerro Frío. It is located at an altitude of 1,785 m between the parallels 18º20′30′ N and 99º17′21′ W, and it encompasses approximately 500 ha [42] (Figure 1).

The settlement is located in a transitional area between tropical deciduous forest and an oak forest [43]. Community surrounding areas include modified environments such as home gardens and farming lands. The dominant climate is semi-warm, semi-humid, with rains during the summer. The total annual rainfall is 924.3 mm and the average annual temperature is 28°C [44].

Inhabitants of El Salto are mestizo. The community was founded by farmers from the southern state of Morelos and by some migrant communities and adjoining villages of the state of Guerrero during the Mexican Revolution (1910–1920). The community is made up of 108 residents that belong to 25 households and each household houses around five people. All families share kinship networks with others, so there is a core of coexistence and knowledge originated from this cause. There are 55 women and 53 men, but children and young people (between 1 and 25 years of age) outnumber adults (60%). Information of 41 inhabitants (21 women and 20 men), ranging between 30 and 70 years of age (average ± SD: 51.4 ± 16.3 years), indicates that about 20% of the informants were born elsewhere, but they had lived at the locality for more than 40 ± 8.6 years. More than three fourths of the informants had finished primary education but 21.9% were illiterate. The festivities in the locality have a religious (mostly Catholic) and civic (school activities) character. The social organization is ruled by community assembly, whose highest authority is the Major’s Assistant.

The main economic activity, practiced by 85.3% of the informants, was subsistence agriculture. Main crops include corn (*Zea mays* L.), bean (*Phaseolus vulgaris* L.) and squash (*Cucurbita argyrosperma* subsp. *argyrosperma*) in an integrated system. Subsistence agriculture was complemented by a range of activities related to the appropriation of natural resources, such as gathering and hunting (4.8%), production of alcoholic beverages such as *mezcal* (made from *Agave angustifolia* Haw.) and “wine” (made from *Vitis tiliifolia* Humb. & Bonpl. ex Roem. & Schult.) (2.4%) and of products derived from cattle, such as milk and cheese (9.7%).

As much as 70.3% of the sample received economic support from the government, this support represented about 20% of their total income and therefore is an important complement to their main economic activities. The average monthly earnings per capita estimated for 2007 were $USD 133.98 ± 57.57. Nevertheless, 44% of the people were exclusively living on remittances coming from the US. Rates of youth migration to the US, mainly after the young people have completed their high-school, are high.

### Ethnobotanical and socio-economic information

Between September 2005 and December 2006, we conducted open-ended and semi-structured interviews [45,46] with all the household heads (n = 41: 21 women and 20 men). To avoid overestimating the knowledge about plants that might have been acquired elsewhere, we selected people older than 30 years old e.g., [47] who had been living in the study site for a minimum of 30 years [48,49]. Interviews were carried out individually to prevent distortions due to the presence of a third person [48,49]. The interviews focused on two aspects a) determining their knowledge of the plant resources (wild, cultivated and weedy species) in their community and b) characterizing the socio-economic conditions of the person being interviewed.

First, to quantify the degree of knowledge of plant resources and to identify the use value of each plant species, we conducted free listings by category of use [46]. The survey only included the theoretical dimension of ethnobotanical knowledge *sensu*[50]. Women’s and men’s knowledge of the plant richness of their community was defined as the number of species mentioned at the time of the interview. Second, we also asked about the following socio-economic information: 1) age, 2) gender, 3) education level (with or without primary studies); 4) origin (local or migrant), 5) main productive activities (farming or stockbreeding), 6) monthly expenditures, as a proxy for monetary earnings, 7) amount received in remittances, and 8) other economic activities (related to temporary earnings perceived as support from governmental institutional projects).

### Data analysis

To determine the use-value of the local flora, we calculated the use-value (UV) index using the algorithm proposed by Phillips and Gentry [21,30], modified by Rossato *et al.*[51] and Lucena *et al.*[52]. The calculation was obtained by counting all the uses mentioned by every person for a specific plant and dividing the result by the total number of informants. The use-value corresponds to the average use associated to each species in a specific community:

$${}_{\mathrm{UVs}=\sum \mathrm{Uis}/n}$$

where *U*_*is*_ is the number of uses mentioned by an informant *i*, for each species *s* and *n* is the number of informants interviewed for each species.

We classified the local environment into five habitats: tropical deciduous forest, oak forest, riparian vegetation, home gardens and farming lands. The first three habitats correspond to vegetation types [43] and the other two to modified environments [53]. We visited each habitat repeatedly, with a different informant each time, to photograph and inventorying useful plants mentioned during the interviews and free listings. Subsequently, in a group meeting using an image projector, a photograph of each plant was showed to all interviewees to reach a consensus and verify that this was the correct etno-specie and avoid confusion by popular synonyms [48,49]. Then, after the identification of plant species was completed, we estimated the richness as the number of plant species per use category per habitat. The habitat of one species was determined based on Rzedowski [43]. The identification of botanical material was carried out at the HUMO Herbarium of the Universidad Autónoma del Estado de Morelos and with experts from the MEXU Herbarium of the Universidad Nacional Autónoma de Mexico. Voucher specimens were deposited at the HUMO and the folio number for each species is shown in (Table 1).

**Table 1:** Ethnofloral listing of El Salto

**Families**	**Scientific name**	**Common name**	**Life forms**	**Uses categories**	**Vegetal structure used**	**Habitat**	**Management**	**Folio number**	**Use value**
FABACEAE	*Lysiloma acapulcense* (Kunth) Benth.	Tepehuaje	A	1, 2, 4, 9, 10 y 12	St, Co and Ro	Tdf	Wi	26268	2,59
FAGACEAE	*Quercus magnoliifolia* Née	Encino amarillo	A	1, 9, 10 y 12	St and Ro	Of	Wi	26177	2,54
FABACEAE	*Eysenhardtia polystachya* (Ortega) Sarg.	Palo dulce o Coatle	A	1, 9, 10 y 12	St, Co and Ro	Tdf	Wi	26209	1,95
CUPRESSACEAE	*Juniperus flaccida* Schltdl.	Cedro	A	2, 4, 8 y 9	St, Fr and Ro	Of	Wi	26223	1,37
ROSACEAE	*Rosa chinensis* Jacq.	Rosa	Sh	5	Fl	Hg	Gr	26286	1,37
MYRTACEAE	*Psidium guajava* L.	Guayaba	A	1, 2 y 3	St, Lf, Co, Fr and Ro	Bg	Si	26187	1,29
FAGACEAE	*Quercus castanea* Née	Encino roble	A	1, 4, 8, 9, 10 y 12	St, Co and Ro	Of	Wi	26176	1,15
SAPINDACEAE	*Ungnadia* sp.	Asicincle	A	2, 4, 9, 10 y 12	St and Ro	Tdf	Wi	26179	1,05
ORCHIDACEAE	*Laelia autumnalis* (La Llave & Lex.) Lindl.	Catarina, Flor de San Diego	He	7 y 11	Cp and Fl	Of	Wi	26161	1,02
CUCURBITACEAE	*Cucurbita pepo* L.	Calabaza	He	3	Fr and Fl	Fl	Gr	26290	1,00
ERICACEAE	*Arbutus xalapensis* Kunth	Madroño	A	3 y 10	St, Ro and Fr	Of	Wi	26110	1,00
FABACEAE	*Mimosa lacerata* Rose	Tecolhuixtle	Sh	4, 9 y 12	St	Tdf	Wi	26274	1,00
FABACEAE	*Phaseolus vulgaris* L.	Frijol	He	3	Fr	Fl	Gr	26291	1,00
SOLANACEAE	*Capsicum annuum* L.	Chile	He	3	Fr	Fl	Gr	26293	1,00
BURSERACEAE	*Bursera fagaroides* (Kunth) Engl.	Copal	A	9	St	Tdf	Wi	26265	0,93
ASTERACEAE	*Tagetes erecta* L.	Cempaxúchitl	He	5 y 7	Cp	Hg	Gr	26259	0,88
SAPINDACEAE	*Dodonaea viscosa* Jacq.	Chapulixtle	A	1, 4, 9 10 y 12	St, Lf and Br	Of	Wi	26217	0,88
ASTERACEAE	*Senecio salignus* DC.	Jarilla	Sh	7	Cp	Of	Wi	26246	0,85
FABACEAE	*Harpalyce arborescens* A. Gray.	Cahuira	A	1, 4, 9 y 12	St, Lf and Co	Tdf	Wi	26245	0,85
LAURACEAE	*Nectandra globosa* (Aubl.) Mez	Aile o Aguacatillo	A	4, 9, 10 y 12	St and Br	Of	Wi	26269	0,85
POACEAE	*Zea mays* L.	Maíz	He	1 y 3	Fr, Bra and In	Fl	Gr	26292	0,85
RUTACEAE	*Ruta chalepensis* L.	Ruda	He	1 y 7	Cp	Hg	Gr	26183	0,85
ASTERACEAE	*Tagetes lunulata* Ortega	Flor de muerto	He	7 y 8	Cp	Tdf	Wi	26185	0,80
MALVACEAE	*Hibiscus rosa-sinensis* L.	Tulipán	Sh	5	Fl	Hu	Cu	26284	0,73
PIPERACEAE	*Peperomia campylotropa* A.W. Hill	Cilantro de peña	He	3 y 11	Cp	Of	Wi	26231	0,73
FABACEAE	*Senna skinneri* (Benth.) H.S. Irwin & Barneby	Paraca	A	1, 8 y 10	St, Fr, Co, Se and Ro	Tdf	Wi	26221	0,71
ANACARDIACEAE	*Spondias mombin* L.	Ciruela	A	3	Fr	Hg	Gr	26113	0,68
FABACEAE	*Conzattia multiflora* (B.L. Rob.) Standl.	Guayacán	A	1, 9 y 12	St and Se	Tdf	Wi	26258	0,68
MALPIGHIACEAE	*Malpighia mexicana* A. Juss	Guajocote	Sh	3	Fr and Co	Hg	Gr	26208	0,63
LAMIACEAE	*Ocimum basilicum* L.	Albahacar	He	1	Cp	Hg	Gr	26182	0,61
ASTERACEAE	*Artemisia ludoviciana* Nutt.	Estafiate	He	1 y 7	Cp	Hg	Gr	26181	0,59
BALSAMINACEAE	*Impatiens balsamina* L.	Chinos	He	5	Fl	Hg	Gr	26278	0,56
FABACEAE	*Leucaena macrophylla* Benth.	Zacahuaje	A	3 y 10	St, Lf, Fl, Fr and Ro	Tdf	Gr	26247	0,56
ASTERACEAE	*Dahlia coccinea* Cav.	Dalia de campo	Sh	5 y 8	Fl and Ro	Tdf	Wi	26153	0,54
FAGACEAE	*Quercus glaucoides* M. Martens & Galeotti	Chaparro	A	8 y 10	St, Lf, Fr and Br	Of	Wi	26255	0,51
ASTERACEAE	*Porophyllum macrocephalum* DC.	Pápalos o Pepichas	He	3	Cp	Tdf	Wi	26119	0,49
PORTULACACEAE	*Portulaca oleracea* L.	Verdolaga	He	3	Cp	Tdf	Wi	26271	0,49
STERCULIACEAE	*Guazuma ulmifolia* Lam.	Cahuilote	A	1, 2, 8, 9, 10 y 12	St, Fr, Lf and Br	Tdf	Wi	26194	0,49
GERANIACEAE	*Pelargonium domesticum* L.H. Bailey	Geranio	He	5	Fl	Hg	Gr	26279	0,46
VERBENACEAE	*Vitex mollis* H.B.K.	Cuayotomate	A	1 y 8	Lf, Fr and Co	Tdf	Wi	26244	0,39
APOCYNACEAE	*Stemmadenia obovata* K. Schum.	Tepechicle	A	2	Br	Tdf	Wi	26211	0,37
APOCYNACEAE	*Plumeria rubra* L.	Rosal	A	7	Cp	Tdf	Wi	26195	0,34
IRIDIACEAE	*Gladiolus grandiflorus* Andrews	Gladiola	He	5	Fl	Hg	Gr	26282	0,29
POLEMONIACEAE	*Loeselia mexicana* (Lam.) Brand	Espinosilla	He	1	Cp	Of	Wi	26165	0,27
FABACEAE	*Acacia angustissima* (Mill.) Kuntze	Timbre	A	1	Br, Co and Se	Of	Wi	26151	0,24
FABACEAE	*Mimosa benthamii* J.F. Macbr.	Tehuixtle	A	4 y 9	St	Tdf	Wi	26210	0,24
AGAVACEAE	*Agave inaequidens* K. Koch	Maguey	He	3 y 4	In	Of	Wi	26133	0,22
JUGLANDACEAE	*Juglans regia* L.	Nogal	A	3, 4 y 9	St and Fr	Gf	Wi	26273	0,22
ULMACEAE	*Celtis caudata* Planch.	Estribillo	A	12	St	Tdf	Wi	26159	0,22
ACANTHACEAE	*Ruellia megasphaera* Lindau	Té negro	He	1	Lf	Hg	Gr	26239	0,20
ASTERACEAE	*Matricaria recutita* L.	Manzanilla	He	1	Cp	Hg	Gr	26285	0,20
MALPIGHIACEAE	*Byrsonima crassifolia* (L.) Kunth	Nanche	A	1 y 3	Fr and Fl	Tdf	Wi	26275	0,20
BIGNONIACEAE	*Tecoma stans* (L.) Juss. ex Kunth	Iztamaxuchil o tronadora	A	1 y 12	St and Cp	Tdf	Wi	26220	0,17
BURSERACEAE	*Bursera ariensis* (Kunth) McVaugh & Rzed.	Cuajiote	A	9	St	Tdf	Wi	26222	0,17
STERCULIACEAE	*Waltheria americana* L.	Manrubio	He	1	Cp	Tdf	Wi	26160	0,17
AMARANTHACEAE	*Amaranthus hybridus* L.	Quintonil	He	3	Cp	Tdf	Wi	26146	0,15
CACTACEAE	*Opuntia ficus-indica* (L.) Mill*.*	Nopal	Sh	1 y 3	Cl and Fr	Tdf	Wi	26240	0,15
LAMIACEAE	*Salvia sessei* Benth.	Vara de San Miguel	Sh	10	St and Br	Of	Wi	26236	0,15
MELIACEAE	*Swietenia humilis* Zucc.	Palo quesero o Palo del zopilote	A	1	Co	Tdf	Wi	26212	0,15
NYCTAGINACEAE	*Bougainvillea glabra* Choisy	Bugambilia	Sh	5	Fl	Hg	Gr	26288	0,15
TAXODIACEAE	*Taxodium mucronatum* Ten.	Sabino	A	4	St	Gf	Wi	26270	0,15
ANACARDIACEAE	*Amphipterygium adstringens* (Schltdl.) Standl.	Cuachalalate	A	1	Co	Tdf	Wi	26180	0,12
ASTERACEAE	*Viguiera sphaerocephala* (DC.) Hemsl.	Ocotillo	Sh	9, 10 y 12	St and Br	Of	Wi	26117	0,12
ASTERACEAE	*Tagetes lucida* Cav.	Pericón	He	1, 7 y 11	Cp, Br, Fl, Co and Se	Of	Wi	26262	0,12
FABACEAE	*Leucaena esculenta* (Moc. & Sessé ex DC.) Benth.	Guaje colorado	A	3	Fl and Fr	Hg	Gr	26219	0,12
LAMIACEAE	*Salvia coccinea* Buc'hoz ex Etl.	Mirto	He	1	Lf	Hg	Gr	26287	0,12
ASTERACEAE	*Vernonia alamanii* DC.	Varaclacote	Sh	12	St	Tdf	Wi	26131	0,10
ASTERACEAE	*Montanoa arborescens* DC.	Vara blanca	He	5, 7 y 9	Cp, St, Fl and Br	Tdf	Wi	26186	0,10
BURSERACEAE	*Bursera bipinnata* (DC.) Engl.	Copal chino	A	1, 2, 4 y 9	St, Br and Lf	Tdf	Wi	26251	0,10
CHENOPODIACEAE	*Teloxys ambrosioides* (L.) W.A. Weber	Epazote	He	3 y 7	Cp	Hg	Gr	26136	0,10
FABACEAE	*Inga vera* Willd.	Cajinicuil	A	3 y 10	St, Fr and Br	Gf	Wi	26152	0,10
SAPINDACEAE	*Serjania triquetra* Radlk.	Bejuco de tres costillas	Cl	1	Cp	Tdf	Wi	26166	0,10
ANACARDIACEAE	*Spondias purpurea* L.	Ciruela de venado	A	3	Fr	Tdf	Gr	26266	0,07
ASTERACEAE	*Tagetes patula* L.	Flor de clemole	He	5 y 7	Cp and Fl	Hg	Gr	26207	0,07
ASTERACEAE	*Calea ternifolia* Kunth var. *ternifolia*	Prodigiosa	He	1	Cp	Tdf	Wi	26154	0,07
EUPHORBIACEAE	*Euphorbia schlechtendalii* Boiss.	Lechecillo	A	1	La	Of	Wi	26193	0,07
FABACEAE	*Erythrina breviflora* Sessé & Moc. ex DC.	Colorín	Sh	3	Fl	Tdf	Wi	26191	0,07
LAMIACEAE	*Salvia leucantha* Cav.	Salvia	He	7	Cp	Of	Wi	26254	0,07
LAURACEAE	*Nectandra salicifolia* (Kunth) Nees	Aguacachil	A	1	Lf and Co	Gf	Wi	26163	0,07
RUBIACEAE	*Randia tetracantha* (Cav.) DC.	Caca de zorra o tecolosapo	A	1 y 3	Fr and Co	Tdf	Wi	26197	0,07
ANACARDIACEAE	*Comocladia engleriana* Loes.	Teclate	A	6	Cp	Tdf	Wi	26205	0,05
APOCYNACEAE	*Mandevilla foliosa* (Müll. Arg.) Hemsl.	Hierba de la cucaracha	He	1 y 6	Cp	Tdf	Wi	26243	0,05
APOCYNACEAE	*Cascabela thevetioides* (Kunth) Lippold	Yoyote	A	2, 7 y 8	Fr and St	Tdf	Wi	26203	0,05
ARACEAE	*Zantedeschia aethiopica* (L.) Spreng.	Alcatraz	He	5	Fl	Hg	Gr	26276	0,05
ASTERACEAE	*Acourtia turbinata* (Lex.) DC.	Cola de coyote	Sh	1	Lf	Fl	Wi	26168	0,05
ASTERACEAE	*Stevia connata* Lag.	Pericón blanco	He	2 y 7	Cp and Fl	Of	Wi	26169	0,05
ASTERACEAE	*Artemisia absinthium* L.	Ajenjo	He	1	Cp	Hg	Gr	26277	0,05
ASTERACEAE	*Bidens odorata* Cav.	Mozote	He	8 y 10	Cp and Fl	Tdf	Wi	26138	0,05
BIGNONIACEAE	*Crescentia alata* Kunth	Cirian	A	1	Fr	Tdf	Wi	26124	0,05
BOMBACACEAE	*Pseudobombax ellipticum* (Kunth) Dugand	Clavellina	A	2 y 8	St and Fl	Tdf	Wi	26157	0,05
BORAGINACEAE	*Cordia morelosana* Standl.	Palo prieto	A	1	Fl	Tdf	Wi	26263	0,05
BRASSICACEAE	*Lepidium virginicum* L.	Mexixi	He	1	Cp	Tdf	Wi	26162	0,05
BURSERACEAE	*Bursera linanoe* (La Llave) Rzed., Calderón & Medina	Copal agüado	A	2, 8 y 9	St and Lf	Tdf	Wi	26242	0,05
CACTACEAE	*Mammillaria nunezii* (Britton & Rose) Orcutt	Rodilla	He	3 y 5	Fr and Fl	Of	Wi	26149	0,05
CAPPARIDACEAE	*Cleome speciosa* Raf.	Barbas de conejo	He	5	Fl	Fl	Wi	26253	0,05
CARICACEAE	*Carica papaya* L.	Papayo	A	1 y 3	Fr and Lf	Hg	Gr	26283	0,05
CARICACEAE	*Jacaratia mexicana* A. DC*.*	Bonete	A	3	Fr and Br	Tdf	Wi	26199	0,05
CRASSULACEAE	*Sedum corynephyllum* Fröd.	Dedito de niño	He	1	Lf	Hg	Gr	26280	0,05
EUPHORBIACEAE	*Euphorbia fulva* Staff	Pegahueso	A	1 y 2	Br and La	Tdf	Wi	26218	0,05
EUPHORBIACEAE	*Jatropha curcas* L.	Mala mujer o Tepechicle	A	1 y 3	La	Of	Wi	26190	0,05
FABACEAE	*Acacia pennatula* (Schltdl. & Cham.) Benth.	Espino blanco	A	8	Co and Fr	Of	Wi	26289	0,05
FABACEAE	*Lysiloma divaricata* Benth.	Mezquite	A	9 y 10	St and Br	Tdf	Wi	26184	0,05
LAURACEAE	*Litsea glaucescens* Kunth	Laurel	He	3 y 7	Cp and Lf	Hg	Wi	26272	0,05
LORANTHACEAE	*Psittacanthus calyculatus* (DC.) G. Don	Injerto de huizache	Sh	1	Lf	Tdf	Wi	26196	0,05
MALVACEAE	*Malva rotundifolia* L.	Malva	He	1 y 3	Cp	Tdf	Wi	26147	0,05
OPILIACEAE	*Agonandra racemosa* (DC.) Standl.	Chicharroncillo	A	1	Lf	Tdf	Wi	26216	0,05
OXALIDACEAE	*Oxalis latifolia* Kunth	Chucuyul	He	3	St	Of	Wi	26230	0,05
ROSACEAE	*Crataegus pubescens* (C. Presl) C. Presl	Tejocote	A	3	Fr	Of	Gr	26232	0,05
ROSACEAE	*Eriobotrya japonica* (Thunb.) Lindl.	Níspero	A	1 y 3	Fr and Lf	Hg	Gr	26189	0,05
ROSACEAE	*Rosa centifolia* L.	Rosa de castilla	Sh	1	Fl	Hg	Wi	26227	0,05
SOLANACEAE	*Solanum lanceolatum* Cav.	Sosa	Sh	2 y 8	Lf and Cp	Tdf	Wi	26198	0,05
TILIACEAE	*Heliocarpus terebinthinaceus* (DC.) Hochr.	Cahuilahua	A	1, 4, 8 y 9	St, Lf, Co and Br	Tdf	Wi	26224	0,05
VERBENACEAE	*Vitex hemsleyi* Briq.	Querengue	A	2, 10 y 12	St and Br	Tdf	Wi	26248	0,05
VERBENACEAE	*Lantana camara* L.	Cinco negritos o Manzanito	Sh	1 y 3	Fr and Lf	Tdf	Wi	26261	0,05
VITACEAE	*Vitis tiliifolia* Humb. & Bonpl. ex Roem. & Schult.	Bejuco de uva	Cl	1, 2 y 3	Fl and Li	Of	Wi	26226	0,05
ACANTHACEAE	*Justicia spicigera* Schltdl.	Muicle	Sh	1	Cp	Hg	Gr	26188	0,02
AGAVACEAE	*Agave angustifolia* Haw.	Agave de mezcal	He	2	In	Of	Wi	26249	0,02
AGAVACEAE	*Agave horrida* Lem. ex Jacobi	Agave de Ixtle	He	2	Bra	Of	Wi	26150	0,02
AGAVACEAE	*Polianthes geminiflora* (Lex.) Rose	Aretito	He	5	Fl	Of	Wi	26174	0,02
ANACARDIACEAE	*Pseudosmodingium perniciosum* (Kunth) Engl.	Cuajiote colorado	A	6	Cp	Tdf	Wi	26225	0,02
APIACEAE	*Eryngium columnare* Hemsl.	Hierba del sapo	He	1	Lf	Of	Wi	26229	0,02
ASCLEPIADACEAE	*Asclepias glaucescens* Kunth	Oreja de liebre	He	1	La	Tdf	Wi	26252	0,02
ASCLEPIADACEAE	*Marsdenia zimapanica* Hemsl.	Pancololote	Cl	3	Fr and La	Tdf	Wi	26241	0,02
ASPHODELACEAE	*Aloe barbadensis* Mill.	Sábila	He	1	Fl and Lf	Hg	Gr	26202	0,02
ASTERACEAE	*Sinclairia glabra* (Hemsl.) Rydb.	Palo Santo o Campozano	A	3 y 9	Fl, St and Br	Tdf	Wi	26126	0,02
ASTERACEAE	*Schkuhria pinnata* (Lam.) Kuntze ex Thell.	Escobita	He	2, 4 y 10	Cp	Tdf	Wi	26139	0,02
ASTERACEAE	*Pectis capillaris* DC.	Limoncillo	He	1	Cp	Of	Wi	26121	0,02
ASTERACEAE	*Cosmos sulphureus* Cav.	Flor amarilla	He	3	Fl	Tdf	Wi	26235	0,02
ASTERACEAE	*Senecio praecox* (Cav.) DC.	Candelerillo	A	5	Fl	Tdf	Wi	26250	0,02
ASTERACEAE	*Calea urticifolia* (Mill.) DC.	Canelilla	Sh	1	Cp	Tdf	Wi	26167	0,02
ASTERACEAE	*Gnaphalium roseum* Kunth	Gordolobo	He	1	Cp	Of	Wi	26256	0,02
ASTERACEAE	*Laennecia filaginoides* DC.	Cimonilla	He	1	Cp	Tdf	Wi	26118	0,02
ASTERACEAE	*Psacalium megaphyllum* (B.L. Rob. & Greenm.) Rydb.	Churumbelo o Sombrerete	He	1	Br	Of	Wi	26127	0,02
ASTERACEAE	*Taraxacum officinale* F.H. Wigg.	Hierba del golpe	He	1	Cp	Fl	Wi	26172	0,02
ASTERACEAE	*Adenophyllum porophyllum* (Cav.) Hemsl.	Árnica	He	1	Cp	Tdf	Wi	26115	0,02
ASTERACEAE	*Tagetes micrantha* Cav.	Anís	He	3	Cp	Of	Wi	26123	0,02
ASTERACEAE	*Tithonia tubiformis* (Jacq.) Cass.	Acahual	He	7 y 8	Cp	Fl	Wi	26140	0,02
ASTERACEAE	*Verbesina crocata* (Cav.) Less.	Capitaneja	He	1	Lf and St	Tdf	Wi	26145	0,02
BEGONIACEAE	*Begonia gracilis* Kunth	Chucuyul de culebra	He	7	Cp	Of	Wi	26137	0,02
BIGNONIACEAE	*Jacaranda mimosifolia* D. Don.	Jacaranda	A	5	Fl	Hg	Gr	26215	0,02
BOMBACACEAE	*Ceiba aesculifolia* (Kunth) Britten & Baker f.	Pochote	A	1, 8 y 12	St, Fl and Sp	Tdf	Wi	26143	0,02
BORAGINACEAE	*Tournefortia hirsutissima* L.	Tlalchinol	He	1	Cp	Fl	Wi	26132	0,02
BURSERACEAE	*Bursera bicolor* (Willd. ex Schltdl.) Engl.	Ticumaca	A	1 y 4	St and La	Tdf	Wi	26214	0,02
BURSERACEAE	*Bursera copallifera* (DC.) Bullock	Copal tieso o Copal seco	A	2, 4, 9 y 12	St	Tdf	Wi	26264	0,02
BURSERACEAE	*Bursera grandifolia* (Schltdl.) Engl.	Palo mulato	A	1	Co	Tdf	Wi	26200	0,02
CALOCHORTACEAE	*Calochortus barbatus* (Kunth) J.H. Painter	Campanita	He	7	Cp	Of	Wi	26228	0,02
COCHLOSPERMACEAE	*Cochlospermum vitifolium* (Willd.) Spreng.	Panicua	A	1	St	Tdf	Wi	26206	0,02
COMMELINACEAE	*Tradescantia commelinoides* Schult. & Schult. f.	Lluvia	He	1	Cp	Hg	Gr	26111	0,02
CONVOLVULACEAE	*Ipomoea murucoides* Roem. & Schult.	Cazahuate prieto	A	1, 3, 8 , 10 y 11	St, Fl, Co and Br	Tdf	Wi	26141	0,02
CONVOLVULACEAE	*Ipomoea pauciflora* M. Martens & Galeotti	Cazahuate blanco	A	8 y 10	St, Fl and Br	Tdf	Wi	26237	0,02
CONVOLVULACEAE	*Ipomoea purpurea* (L.) Roth	Quiebra plato	Cl	5	Fl	Hg	Wi	26142	0,02
CRASSULACEAE	*Kalanchoe pinnata* (Lam.) Pers.	Orejona	He	1	Lf	Hg	Gr	26164	0,02
CRASSULACEAE	*Echeveria obtusifolia* Rose	Siempreviva	He	1	Fl	Of	Wi	26173	0,02
CRASSULACEAE	*Sedum oxypetalum* Kunth	Cuajiote de peña	Sh	1	Lf and St	Of	Wi	26122	0,02
CUCURBITACEAE	*Sechium edule* (Jacq.) Sw.	Chayote	Cl	1 y 3	Fr and Lf	Hg	Gr	26238	0,02
EUPHORBIACEAE	*Euphorbia calyculata* Kunth	Coralillo	A	6	Cp	Tdf	Wi	26129	0,02
EUPHORBIACEAE	*Ricinus communis* L.	Higuerillo	He	1	Lf	Fl	Wi	26109	0,02
FABACEAE	*Canavalia villosa* Benth.	Flor de gallito	Cl	3	Fl	Of	Wi	26120	0,02
FABACEAE	*Calliandra grandiflora* (L'Hér.) Benth.	Cabellito de ángel	He	1 y 3	Fl and Br	Of	Wi	26204	0,02
FABACEAE	*Marina scopa* Barneby	Escoba colorada	Sh	4	St	Tdf	Wi	26135	0,02
FABACEAE	*Phaseolus leptostachyus* Benth.	Chinela	He	3	Bu	Tdf	Wi	26171	0,02
FABACEAE	*Senna hirsuta* (L.) H.S. Irwin & Barneby	Carnizuelo	He	1	Lf and Fl	Tdf	Wi	26260	0,02
FABACEAE	*Zornia thymifolia* Kunth	Sangrinaria o Cascabelilllo	He	1	Cp	Of	Wi	26155	0,02
FABACEAE	*Crotalaria cajanifolia* Kunth	Crotalaria	He	3	Fl	Tdf	Wi	26128	0,02
FABACEAE	*Haematoxylum brasiletto* H. Karst.	Palo de brasil	A	1	Co	Tdf	Wi	26134	0,02
FABACEAE	*Pithecellobium dulce* (Roxb.) Benth.	Guamúchil	A	3, 9 y 10	St, Fr and Br	Fl	Wi	26213	0,02
FLACOURTIACEAE	*Xylosma flexuosa* (Kunth) Hemsl.	Abrojo	A	2 y 4	St	Of	Wi	26130	0,02
IRIDIACEAE	*Tigridia multiflora* (Baker) Ravenna	Gallito o Aretito	He	7	Cp	Of	Wi	26233	0,02
LAMIACEAE	*Salvia microphylla* Kunth	Hierba del golpe	He	1	Lf	Fl	Wi	26178	0,02
LAMIACEAE	*Mentha piperita* L.	Hierbabuena	He	1 y 3	Cp	Hg	Gr	26281	0,02
LILIACEAE	*Bessera elegans* Schult. f.	Aretito	He	7	Cp	Of	Wi	26175	0,02
LOGANIACEAE	*Buddleja americana* L.	Lengua de vaca	He	1	Lf	Fl	Wi	26116	0,02
MALPIGHIACEAE	*Galphimia glauca* Cav.	Vara de San Agustín o Flor de Santa Teresa	Sh	1	Cp	Of	Wi	26114	0,02
MALPIGHIACEAE	*Bunchosia canescens* (W.T. Aiton) DC.	Nanche de perro	Sh	1	Cl, Fl and Lf	Hg	Gr	26112	0,02
MORACEAE	*Ficus cotinifolia* Kunth	Cabrigo	A	8	Fr	Gf	Wi	26267	0,02
ORCHIDACEAE	*Stenorrhynchos lanceolatus* (Audl.) Rich.	Espiguita	He	5	Fl	Of	Wi	26144	0,02
PASSIFLORACEAE	*Passiflora edulis* Sims	Maracuya	Cl	3	Fr	Hg	Gr	26148	0,02
PASSIFLORACEAE	*Passiflora foetida* L.	Granada	Cl	3	Fr	Tdf	Wi	26170	0,02
RUBIACEAE	*Galium mexicanum* Kunth	Pegarropa	He	1	Cp	Tdf	Wi	26192	0,02
SCROPHULARIACEAE	*Castilleja arvensis* Schltdl. & Cham.	Tornillo o cola de borrego	He	1	Cp	Fl	Wi	26234	0,02
SELAGINELLACEAE	*Selaginella lepidophylla* (Hook. & Grev.) Spring	Flor de piedra	He	1	Cp	Of	Wi	26156	0,02
SOLANACEAE	*Datura stramonium* L.	Toloache	He	1 y 7	Lf and Fl	Fl	Wi	26257	0,02
SOLANACEAE	*Nicotiana tabacum* L.	Tenejiate	He	2	Lf	Fl	Wi	26158	0,02
VERBENACEAE	Priva mexicana (L.) Pers.	Hierba del cáncer	He	1	Cp	Tdf	Wi	26125	0,02
VERBENACEAE	*Verbena carolina* L.	Verbena	He	1	Cp	Of	Wi	26201	0,02

Life forms, A: Arboreal; Sh: Shrubby; He: Herbaceous; Cl: Climbing. Use categories, 1: Medicinal; 2: Crafts; 3: Edible; 4: Domestic wooden tools; 5: Ornamental; 6: Poison; 7: Mystical-religious; 8: Fodder; 9: Timber yielding-construction; 10: Firewood; 11: Commercialization of wild plants; 12: Farming wooden tools. Vegetal structure used: Fr: Fruit; Complete plant, Cp; Lf: Leaf; St: Stem; Br: Branch; In :Inflorescence; Fl: Flower; Bu: Bulb; Ro: Root; La: Latex; Bra: Bracts; Co: Cortex; Se: Seed; Sp: Spicules; Cl: Cladodium; Li: Liana. Habitat refers to the location where the species was collected and/or where local settlers gather it, Tdf: Tropical deciduous forest; Of: Oak forest; Hg: Home gardens; Fl: Farming lands; Gf: Gallery forest. Management, Wi: Wild; Gr: Grown. Use-value 0 “zero” corresponds specifically to species mentioned by local guides but not quantified in the interviews.

To determine the association of socio-economic factors and ethnobotanical knowledge, as measured in the described interviews, we used a non-metric multidimensional scaling analysis (NMDS). This technique is appropriate for non-normal discontinuous data, such as the data used in this study. In our analysis, answers from the interviews were used as external variables to interpret ordination. Both the continuous variables (i.e., remittances, age, monthly expenditures), and the nominal variables, (i.e., main productive activities, education level, gender, origin, and other economic activities -earnings from a governmental source and origin of the person) were adjusted on the ordination. Two matrices were used to carry out the multivariate analysis. The main matrix included the useful species registered during open-ended and semi-structured interviews. The variables appear in rows and the interviewed people in columns. The secondary matrix included the socio-economic factors in rows and the interviewed people in columns.

The result from the analysis allowed for the representation of vectors as arrows that point in the direction towards which the variable being assessed changes the most; this is called direction of the gradient. The arrow’s length is proportional to the correlation between the ordination and the assessed variable, and it is called force of the gradient. The analysis estimates a value for r^2^, which represents the goodness of fit of the vector. Significance or p-value is based on the random permutations of the data. In the case of the factors, the analysis also estimates a value for r^2^ as goodness of fit, and a p-value that allows to test the significance of the factor on the ordination. The graphic representation included the main groups’ centroids. Only the vectors and factors that turned out to be significant (p < 0.05) were graphically represented. The “Vegan” module [54] from the R program [55] was used for the statistical analyses. The difference in terms of the knowledge of the number of species between the genders was assessed for the six main use categories (Table 1).

Paired t-tests by household and a Wilcoxon signed rank test were used to determine if there were differences between men and women in terms of the number of species they knew by use category and in terms of the total number of species known. The paired difference distribution of paired t-test was analyzed by means of the Shapiro–Wilk’s W normality tests.

## Results

### Knowledge of plant species

A total of 185 species, belonging to 149 genera and 69 families (Table 1), were recorded. We distributed those species in 12 use categories. We found a total of 310 different uses for the 185 species; thus, the richness of use is greater than the richness of species since some of the species had more than one use (Table 2). The richest families were Asteraceae and Fabaceae and the richest genera were *Bursera* and *Tagetes*.

**Table 2:** Use diversity among the 185 species and distribution in the five studied habitats

**Use categories**	**Tropical deciduous forest**	**Oak forest**	**Riparian vegetation**	**Home gardens**	**Farming lands**	**Number of species**^**1**^	**Percentage**^**1**^
Medicinal	40	20	2	18	10	90	29
Edible	19	11	3	10	4	48	15.5
Timber yielding-construction	17	6	1	0	1	25	8.1
Firewood	12	8	1	0	1	22	7.1
Crafts	13	6	1	0	2	22	7.1
Mystical-religious	4	9	0	6	2	21	6.8
Fodder	14	3	1	0	1	19	6.1
Farming wooden tools	13	5	0	0	0	18	5.8
Domestic wooden tools	11	6	2	0	0	19	6.1
Ornamental	3	3	0	11	1	18	5.8
Poison	4	0	0	0	0	4	1.3
Commercialization of wild plants	1	3	0	0	0	4	1.3
Total	151	80	11	45	23	310	100

^1^Includes species with more than one use (n = 310 uses).

According to their life form, the greatest proportion of useful plants registered at the locality included herbaceous (47%), arboreal (38%), shrubby (11%) and climbing (4%) species. The most used plant structures comprised the stem (28%) and the complete plant (21%), but there is no consistent pattern across the use categories (Table 1). The distribution of the richness of species use per habitat was consistent across habitat only in the case of the medicinal and edible species, the two most common uses. The ranking arrangement of the other use categories changed according to the habitat; this could be related to the availability of the species in each habitat (Table 2).

Tropical deciduous forest was the habitat with the highest proportion of useful species known to the sample, as it is shown by the greatest richness value of the useful plants and the greater diversity of uses for this habitat (Tables 2 and 3).

**Table 3:** Knowledge of plant species at natural and artificial environments in a mestizo community in the center of Mexico

**Habitat**	**Number of species**	**Percentage**
Tropical deciduous forest	80	43.2
Oak forest	47	25.5
Riparian vegetation	6	3.2
Home gardens	35	18.9
Farming lands	17	9.2

### Species use-value

**Figure 2:**
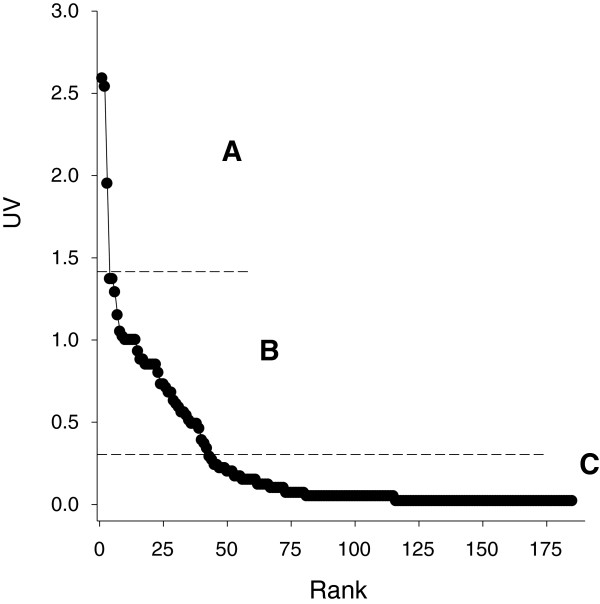
**Rank of use-values of plant species.** The use-values were classified into three groups: **A** (4–6 UV; three species), **B** (0.35-1.37 UV; 39 species) and **C** (0–0.29 UV; 143 species).

We defined three groups according to the species use-values (Figure 2). The first group (*A*) comprises three multi-purpose species (4 to 6 uses), wit use-values >1.5. Species in this group correspond to trees appreciated for their timber: tepehuaje (*Lysiloma acapulcense* (Kunth) Benth; 2,59*),* yellow oak (*Quercus magnoliifolia* Née; 2,54*)* and palo dulce (*Eysenhardtia polystachya* (Ortega) Sarg.; 1,95). The second group (*B*) comprises 39 multi-purpose species with use-values between 0.34 and 1.37. Species in this group range from one (*Rosa chinensis* Jacq.; 1,37) to six uses (*Quercus castanea* Née; 1,15), and many are used for medicinal purposes (11 species), timber (10 species), food (10 species), fuel (9 species), mystical-religious ceremonies (6 species), ornamental purposes (5 species), and commerce (2 species). The last group (*C*) comprises 143 species with use-values between 0 and 0.29, which show a distinct low diversity of uses per species. In fact this group includes only one taxon used for five purposes (*Ipomoea murucoides* Roem. & Schult.; 0,02). Species in this group are mainly used for medicinal needs (76 species), timber (55 species), food (35 species), religious activities (15 species), ornamental and fuel (12 species), and commercial activities (2 species). Four species on the group are toxic.

### Socio-economic variables and their association to ethnobotanical knowledge

**Figure 3:**
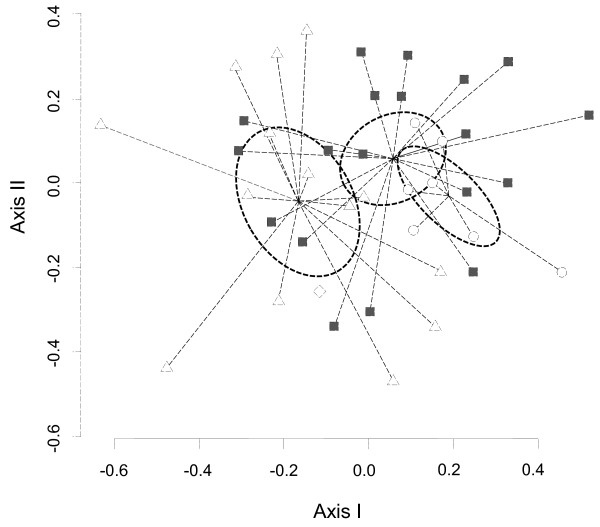
**NMDS ordination of relationship between type of economic activity (stockbreeding, farming, both and others) and knowledge of plant species.** The stockbreeding variable only includes one individual (diamond symbol). The farming group is represented by the set of grays-square symbols. Open circle symbols correspond to farming-stockbreeding practices. The remaining individuals (open triangle symbols) were grouped under others economic activity.

**Figure 4:**
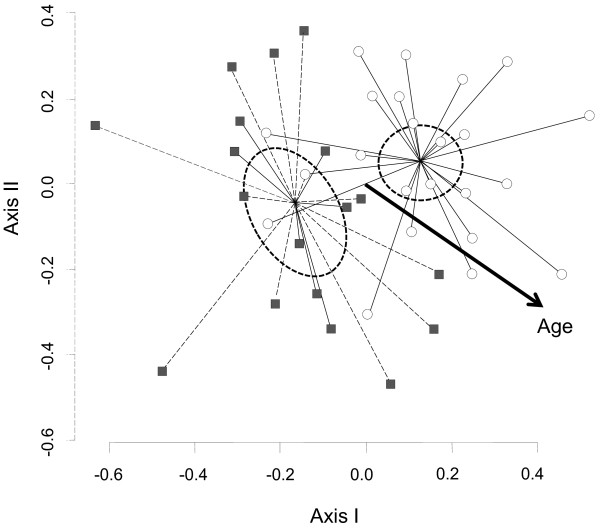
**NMDS ordination of relationship between gender and knowledge of plant species between men and women.** The set of men is constituted by open circles. The group of women is included in the set of grays-square symbols.

Results from the NMDS analysis and subsequent adjustment of socio-economic variables suggested that the variables age, gender, farming and stockbreeding were associated in a statistically significant way to the knowledge of plant resources (Figures 3 and 4, Table 4). In contrasts, education level, origin, and the three others variables related to economic status (i.e., monetary earnings, remittances, governmental monetary support) were not associated to the ethnobotanical knowledge of a person (Table 4).

**Table 4:** Influence of socio-economic variables and factors on ethnobotanical knowledge at El Salto community

**Variables**	**P **^**#**^	**r**^2*^
Age^a^	**0.0204**	0.18
Gender^b^	< **0.001**	0.24
Education level^b^	0.328	0.03
Origin^b^	0.646	0.01
Main productive activities^b^	**0.006**	0.20
Monthly expenditures^a^	0.6986	0.02
Remittances^a^	0.284	0.03
Other economic activities^b^	0.177	0.04

^a^= Vectors; ^b^= Factors; ^#^= P-values based on 1000 permutations (statistical significant p-values are highlighted in bold); ^*^= Coefficient of determination.

The statistical analysis showed a clear spatial separation between two groups due to the differences in terms of men’s and women’s knowledge (Figure 3). Men from El Salto mentioned an average of 53.0 ± 10 (mean ± standard deviation) plant species, whereas women referred to an average of 38.9 ± 6 plant species. The difference was statistically significant (Student’s test, T_15_ = 4.8, p < 0.001). However, when analyzing the indication of plant species according to the general use category, we found that women significantly mentioned more ornamental and mystical-religious plants species than men (Table 5). In contrast, men mentioned significantly more plant species used for building houses or fences, crafts, farming wooden tools, firewood, domestic wooden tools, fodder, poisons, and the commercialization of wild plants than women (Table 5). No differences were found between men’s and women’s responses in relation to edible or medicinal plants (Table 5).

**Table 5:** Results of paired student t-test or Wilcoxon signed rank test (t and W, respectively) of plant species by use category mentioned by men and women at El Salto

**Use category**	**Number of species mentioned by men**	**Number of species mentioned by women**	**Test value**	**P-value**^**#**^
Medicinal	4.1 ± 1.9	4.7 ± 1.9	T = 1.1	P = 0.31
Edible	5.3 ± 1.1	5.2 ± 1.2	W = 22.5	P = 0.53
Timber yielding construction	7.5 ± 1.4	5.1 ± 2.7	T = 2.9	**P = 0.02**
Firewood	3.1 ± 0.9	2.6 ± 0.8	W = 32.5	**P = 0.04**
Crafts	3.5 ± 1.9	0.6 ± 0.9	W = 120	**P < 0.01**
Mystical-religious	7.4 ± 2.0	8.8 ± 1.9	T = 2.1	**P = 0.049**
Fodder	3.6 ± 1.9	0.7 ± 1.1	W = 136	**P < 0.01**
Farming wooden tools	4.3 ± 2.1	1.6 ± 1.8	T = 4.1	**P < 0.01**
Domestic wooden tools	4.0 ± 2.2	2.2 ± 1.4	T = 2.7	**P = 0.02**
Ornamental	2.6 ± 0.8	3.2 ± 0.5	W = 3	**P = 0.04**
Poison	3.8 ± 1.0	1.0 ± 0.0	W = 136	**P < 0.01**
Commercialization of wild plants	3.8 ± 0.9	3.3 ± 0.4	W = 21	**P = 0.03**

^#^= Statistical significant p-values are highlighted in bold.

## Discussion

### Ethnofloristic richness

Species used at El Salto represent only 2.6% of the useful plants previously reported by Caballero and Cortés [9] for peasant –indigenous and mestizo– communities in Mexico. However, if we compare our data with the number of useful species reported by Bye *et al.*[56] for a larger territory comprising 12 ejidos near the region of Chamela, Jalisco, Mexico, the number of useful species at El Salto is 12.4% higher than the number in those ejidos (185 vs. 162 species, respectively). This difference is more remarkable given that the ecological conditions are similar at both sites, suggesting that the size of the site is not the only factor that determines the number of useful species known by its inhabitants. Rather, historical, cultural and socioeconomic traits are essential to understand the local knowledge of useful plant diversity in a particular study area [57].

The plants used at El Salto include some botanical families (Asteraceae, Fabaceae, Burseraceae, Lamiaceae, Verbenaceae, Euphorbiaceae, Anacardiaceae and Solanaceae) which play an important role to satisfy local needs, as well as some considered of uppermost importance at the national, state, and regional levels [9,22,57]. While these families contributed many useful species, the highest percentage of species listed came from a wide range of families, as follows: 7.3% of the families contributed three species each, 17.4% of the families contributed two species each, and 56.5% of the families contributed one species each. The high incidence of new uses of plants reported in the community could be accounted by the occurrence of many rare species (*n =* 136 species), which in turn could be explained by the presence of two major types of vegetation in the area: tropical deciduous and oak forests. In terms of the life forms of the resources, there is a high variation within each use category. Furthermore, our results indicate the preponderance of herbaceous species over other life forms, especially in modified environments (i.e., home gardens and farming lands); this fact is in agreement with the pattern reported for Mexico [9].

When analyzing the use value of plants, we find a predominance of medicinal uses. Moreover, this category is the one that displays more uses per species. This result appears to be constant among mestizo communities in the country, as it has been suggested by Bye [58] on a review of case studies among mestizo and indigenous groups in Mexico. Furthermore, this author has pointed out to differences in terms of the use of alimentary plants among the social groups mentioned: edible plants are of immediate concern for the indigenous societies while they only play a secondary role for mestizos.

We also found that people recognize a greater richness of species and diversity of uses in wild habitats than in modified environments. This finding contrasts with some studies that show that home gardens and farming lands harbor a greater biological diversity than the one registered in the wild environment [53,59]. Altieri *et al.*[53] show that tropical agroecosystems can contain more than 100 species; whereas Pulido *et al.*[59] point out that a family orchard with an average extension of 0.5-2.5 hectares located in the tropical deciduous forest holds a diversity comprising 92 species (61% of them are native to the area). The differences could be methodological, as our study refers to species recognized, whereas the other studies are based on plant inventories. The differences could also be explained by the existence of a direct relation between the relative diversity of species in modified environments and the availability of irrigation water, as was described by Villa and Caballero [60]. Taking into account this relation, we hypothesize that the extended dry season that characterizes the region inhabited by the community of El Salto severely limits the amount of irrigation water, which in turn restricts the diversity of useful plant species that can be maintained in managed environments.

The results from this study reveal the importance that wild ecosystems have for mestizo communities in terms of the development of basic rural subsistence activities in dry tropical areas. However, our research also shows that both, wild habitats and artificial environments, are valuable to understand a group’s ethnobotanical knowledge.

### Species use-values

The highest use-values among species from groups *A* and *B* were registered in relation to timber, typically employed for house-building, firewood, and the manufacturing of farming tools, crafts, and household possessions. At present, these activities are not so frequent among the studied population, given the time and energy needed to manufacture products. However, even though these uses are less frequent, their knowledge persists, a situation that is similar to what Byg and Balslev [15] show for the use of palm species among the Shuar in Ecuador. For example, people maintain a body of knowledge related to timber species differentiating between spongy wood (hollow and brittle), solid wood (useful to manufacture farming tools), or wood that will become “good hot coal”, i.e., firewood that lasts longer ignited [57].

When use-values were analyzed, we found that all of the species included in group *A* (i.e., UV from 2.59 to 1.95) were mentioned by all the inhabitants and all of them were multi-purpose. To us, this finding illustrates the fact that the community has undergone a process of cultural appropriation of the floral diversity.

Even though groups *B* (i.e., UV from 1.37 to 0.34) and *C* (i.e., UV from 0.29 to 0.1) displayed the greatest floral diversity (182 species), there was a variation in terms of the multi-purposefulness and the number of uses was not stable. There was a tendency towards the use of timber yielding species in group *B* and of medicinal species in group *C,* suggesting that group B is probably the most important socially as it comprises the greatest number of species with the highest number of applications.

It is worth noting that none of the medicinal species displayed a high rate of use-value. In other words, the use of the most important plants for the community (i.e., the ones with highest use-value) is not related to the importance ranking by category of dominant use at the site. Such situation could be explained due to the multi-purposefulness that characterizes most species, since their inclusion in different use categories increases their potentiality, in other words, their use-value is enhanced while their exclusiveness for a specific use category decreases. Such speculation calls for a more complex analysis, since it is known that medicinal plants are culturally preeminent among mestizo communities [56].

### Variables associated to knowledge

Our findings suggest that the knowledge of plant resources is associated mainly to socio-economic activities, age and gender, which is consistent with other ethnobotanical investigations [6,12,15,25]. In terms of the socio-economic aspects, despite the fact that occupation, i.e., farming and stockbreeding, was significantly associated to knowledge, it only accounted for 20% of the variation in the ethnobotanical knowledge. This suggests that there are other factors, not included in the analysis, which could influence ethnobotanical knowledge. Such other factors might entail cultural aspects such as ideological structures, ceremonies, significance and classification systems, production techniques and practices [5,8,14,27,37], or ecological ones, which have historically been poorly explored –e.g., density of useful species, floral heterogeneity at the site, dominant biological forms, altitudinal variations, types of vegetation, selective floral and fruit morphology and phenology– [16,22,26,28,52,61,62].

Farming and stockbreeding constitute common activities that seem to provide a particular contribution to ethnobotanical knowledge. Some studies show that conducting primary activities contributes to use and management of natural resources [25,27]. The relation between animal rearing and ethnobotanical knowledge can be explained through several examples in this study. Thus, as livestock rearing constitutes the settlers’ main activity, there is a need for them to know the plant resources that are helpful in the treatment of cattle’s gastrointestinal diseases. This knowledge is based on the observation of the animals’ alimentary habits with respect to wild and fodder plants, as well as on the detection of the toxic species that are eliminated from the environment to avoid that cattle consume them. Ethnobotanical knowledge also allows farmers to use alternative fodder in times of economic shortage or draught and also contributes to livestock health through the prevention of common diseases. Farmers are also familiar with the species that are used to craft farming tools, for house-building activities (with specific traits such as resistance, flexibility, duration and pliability, as living fences and as tutors). Some species are also tolerated due to the benefits they offer such as shade, medicine and food [57].

The informants’ age also seem to be associated with ethnobotanical knowledge. Older people knew more useful plant species than younger people, probably because ethnobotanical knowledge tends to accumulate through the life cycle, as has been found elsewhere [12,15,27,28]. Garro [63] indicates that aging is naturally associate with the process of knowledge acquisition as the pass of time help individuals accumulate knowledge and experience. Furthermore, in some studies, age seem to be the only variable associated with knowledge [64], although some other authors have found no association between age and knowledge [15]. Although most studies highlight knowledge differences between young and old people, our results suggest that ethnobotanical knowledge continues to accumulate after 30 years of age.

We also found differences between men’s and women’s knowledge in relation to the plants they use at the interspecific and the intracategorical levels, as has been pointed out by other authors e.g., [6,19,28]. Women’s knowledge, in terms of the proportion of useful species they know, is closely associated to the treatment of diseases, the use of plants that embellish their household and of those related to rituals. The knowledge displayed by men is more diverse, since it includes a greater number of species used because of their wood quality to produce crafts and farming tools, to build houses, as fuel and household possessions, as well as species used as cattle fodder. Men are also more knowledgeable than women about plants that can cause bodily harm (swelling and irritation) during working days. Working with Raramuri indigenous people, Camou-Guerrero *et al.*[6] found that women had higher knowledge of medicinal and edible plants than men. As other authors [12], we did not find such differences in the community of El Salto. In sum, our results suggest that gendered division of labor within the family has resulted in constant interaction with the resources corresponding to specific activities. This phenomenon has determined how different species have acquired cultural importance for a specific gender in different cultural contexts [2,65].

Other factor, such as the level of formal education, origin, and economic variables (income, remittances, and subsidies) are not associated to ethnobotanical knowledge. Previous research with young people has shown that the level of formal education bears a negative association with ethnobotanical knowledge [66], probably because time invested in schooling deflects from time invested in ethnobotanical knowledge also generating a lack of interest on the environment [26]. In contrast, Godoy [67] notes that formal education can lead to practices of use of more sustainable resources and the “environmental awareness”. In most of the studies it has been found that education is associated with the loss of language and ethnobotanical knowledge in indigenous communities and of mixed origin (mestizo-indigenous) [27]. If the loss of indigenous language is the main factor that drives the loss of ethnobotanical knowledge, this could help explain why in the studied Spanish-speaking mestizo community we do not find the expected negative association between schooling and ethnobotanical knowledge.

We did not find significant differences in ethnobotanical knowledge between people from the area and outsiders. The finding dovetails with what was reported by Byg and Balslev [15], who in their study in Ecuador find no differences between the ethnobotanical knowledge of indigenous peoples and colonists. In our case study, this could be due to the fact that people who have migrated to El Salto are coming from areas with the same type of vegetation and productive activities, and to the fact that most people migrated to the area during childhood.

Income, remittances and subsidies are all economic indicators of family well-being, which have also often been related to the loss of ethnobotanical knowledge, as income allows people to access market goods that substitute plant-made products [15,34]. However, we did not find such a relation. We argue that this could be due to the lack of large differences in the sample. In most cases families depend on remittances and subsidies, which are then invested in primary activities. Reyes-García *et al*. [68] show that conducting forest and farm activities is associated with greater ethnobotanical skills and with greater theoretical ethnobotanical knowledge, even if those are market oriented, thus implying that some forms of economic development can take place without eroding local ecological knowledge.

Although the variables presented have been analyzed independently, they do not act in independent way, or always have a linear relation with ethnobotanical knowledge. The acquisition of ethnobotanical knowledge is a complex process and we can not assure that the variables analyzed are the only direct drivers of the transmission and acquisition of this knowledge. We suggest that the generation of ethnobotanical knowledge should be understood as a dynamic social process, driven by the current interaction with the ecosystem given the importance of multiple socioeconomic and cultural factors [69].

## Conclusions

We found that the ethnobotanical knowledge of a mestizo community settled in a tropical deciduous forest environment is actually larger than the ethnobotanical knowledge reported for other regions in the same environment, which is widely documented in Mexican literature. Thus, it is considered that ethnobotanical research among mestizo populations is essential to detect locations with vegetal and cultural richness in order to build up the implementation of interdisciplinary programs that favor the development of feasible local proposals for biocultural conservation, particularly in cultural strengthening of traditional knowledge systems for an effective forest management.

The use-value rate constitutes a useful tool to approach a group’s socio-economic and cultural expressions, since it allows the most used species as well as tendencies in use. Nevertheless, it is also necessary to assess the species’ frequency of use, since although species might be known and valued, they might not be currently in use. The use-value rate also allows to assess directly the pressure being exerted on a particular species or on a vegetal community.

While the use-value technique proposed by Phillips and Gentry [70] has limitations related to the interpretation of the pressure to use vegetation resources, it is important to note that the use of any natural resource is performed within specific cultural contexts [14,26,62,71]. Studies such as this one, with a focus on the relation between the local lore and native plants, can become important tools for the conservation of tropical resources by establishing management strategies based on local demands and by prioritizing the selection of species in terms of conservation efforts. Therefore, as has been mentioned by Lawrence *et al.*[19], a key challenge for ethnobotanists is to develop effective ways of understanding both people and the value of plants and more particularly of revealing the socio-economic context and the ecological values that influence them.

The knowledge produced during the interaction between plants and peasant societies is diverse and selective, in other words, the wisdom that articulates the use of plant resources varies according to the type of vegetation present in the location, the cultural value of certain plants as well as their economic and social relevance. In this study we found that socio-economic variables, such as those related both to farming/stockbreeding activities and to the differences according to age and gender, have a strong association to the ethnobotanical knowledge. Older people reported more useful plant species and could be an important cultural reservoir of ethnobotanical knowledge in mestizo rural communities. Men’s and women’s ethnobotanical knowledge differed in terms of use categories and of the number of species they recognize; this is expressed by different patterns of cultural appropriation and reproduction concerning the use of certain species. Nevertheless, there is consensus related to the knowledge of medicinal and alimentary plants at the family and community levels.

The use of this type of information could be very valuable for studies directed towards the restoration of ecosystems with species of local importance, particularly if the most valued plant resources known to men and women are taken into account. Identifying these resources could enhance the chances of success as well as the sustainability of silvicultural programs oriented to biological conservation and rural development.

Finally, ethnobotanical knowledge, understood as a dynamic and socially specific process, deserves deeper study to determine its origin, transformation and its possible loss. This will allow assessment and systematization of patterns of plant knowledge, which involves possibilities of political actions in programs to strengthen knowledge and the sociocultural, economic and ecological factors that are related to prevent their erosion. The results of this study contribute to integrate the local knowledge of a mestizo community into appropriate proposals to preserve it.

## Consent

Prior informed consent was orally obtained from all participants, in line with the requirements of the Universidad Autónoma del Estado de Morelos ethical prescriptions for publication of this report and any accompanying images.

## Competing interests

The authors articulate that they have no competing interest.

## Authors’ contributions

LB main author, involved in the study design, conducting of interview, field work, literature review and general data collection and systematization, wrote the first draft and concluded the final version of this paper with the rest of the coauthors. AO and BM main coordinators of the research project; contributed with original ideas and data, and participated in reviewed several drafts of the manuscript. NM suggested and performed statistical analysis, teaming with LB, AO and BM in the review of drafts and in the final version this paper. VR contributed to review drafts of the paper and the final manuscript. All authors read and approved the final manuscript.

## Authors’ information

LB student at the Postgraduate of Botany, Colegio de Postgraduados. AO, NM and BM full time researchers at CIByC, UAEM. VR researcher at the Universitat Autónoma de Barcelona, Spain.
